# Comparison of unilateral biportal endoscopic lumbar fusion and modified minimally invasive tubular lumbar fusion for lumbar disc herniation: a two-year retrospective study

**DOI:** 10.3389/fneur.2025.1729583

**Published:** 2025-12-15

**Authors:** Jialong Qi, Mingxiang Liu, Tao Shan, Zhou Dong, Guosong Han, Zhihao Ni, Ke Zheng, Li Ma, Zhidong Zhang

**Affiliations:** Department of Orthopedics, The Third Affiliated Hospital of Anhui Medical University (The First People's Hospital of Hefei), Anhui Medical University, Hefei, China

**Keywords:** unilateral biportal endoscopic lumbar fusion, modified minimally invasive tubular lumbar fusion, lumbar disc herniation, UBE-TLIF, MIS-TLIF

## Abstract

**Objective:**

To compare the medium- and long-term clinical outcomes of Unilateral Biportal Endoscopic Lumbar Fusion (UBE-TLIF) and Modified Minimally Invasive Tubular Lumbar Fusion (MIS-TLIF) for treating Lumbar Disc Herniation (LDH).

**Methods:**

A retrospective analysis was conducted on 86 patients with single-level LDH who underwent surgery between August 2022 and August 2023. Patients were allocated to two groups: 42 underwent UBE-TLIF and 44 underwent MIS-TLIF. We recorded operative time, postoperative drainage volume, and complication rates. Pain and functional recovery were assessed using the Visual Analogue Scale (VAS) for back and leg pain and the Oswestry Disability Index (ODI) preoperatively and at 3 days, 1, 6, 12, and 24 months postoperatively. Surgical outcomes were evaluated at 12 months using the MacNab criteria.

**Results:**

The mean operative time was significantly longer in the UBE-TLIF group than in the MIS-TLIF group (140.42 ± 16.02 min vs. 92.15 ± 13.14 min, *p* < 0.05). However, the UBE-TLIF group had a significantly lower postoperative drainage volume (65.79 ± 13.46 mL vs. 103.58 ± 12.56 mL, *p* < 0.05). Both groups showed significant improvements in VAS and ODI scores at all postoperative time points compared to preoperative baselines (*p* < 0.05). Although most intergroup differences in VAS and ODI scores were not statistically significant (*p* > 0.05), the UBE-TLIF group demonstrated lower scores across all follow-ups, with the difference at 3 days postoperatively being significant (*p* < 0.05). According to the MacNab criteria, the excellent-good rate was 95.2% for UBE-TLIF and 95.5% for MIS-TLIF, indicating no significant difference (*p* > 0.05). The fusion rates, assessed via the Bridwell grading system, were 90.4% (UBE-TLIF) and 93.2% (MIS-TLIF), which was also not a statistically significant difference (*p* > 0.05).

**Conclusion:**

Both UBE-TLIF and MIS-TLIF demonstrate comparable medium- and long-term clinical efficacy for LDH. UBE-TLIF is associated with less postoperative drainage and significantly better early pain relief, suggesting less soft tissue trauma. However, it was linked to a longer operative time in this study.

## 1 Introduction

With the acceleration of social pace and changes in human lifestyle, the incidence of lumbar degenerative diseases is increasing (1). With the acceleration of social pace and changes in human lifestyle, the incidence of lumbar degenerative diseases is increasing (2). This condition not only impacts patients’ quality of life but also imposes a substantial socio-economic burden.

As demands for quality of life rise, so does the need for surgical treatment of LDH (3). For patients with loss of intervertebral disc height or spinal instability, decompression alone often fails to provide satisfactory long-term outcomes (4). Lumbar interbody fusion remains the gold standard for treating such cases. Since Foley’s initial report (5) on Minimally Invasive Transforaminal Lumbar Interbody Fusion (MIS-TLIF), the technique has gained widespread recognition among surgeons due to its minimal muscle dissection, reduced trauma, and proven clinical efficacy, and is now commonly performed in hospitals at various levels. However, MIS surgery remains a tubular technique performed in an air medium, presenting clinical challenges such as limited visualization and postoperative low back pain. In recent years, driven by the deepening adoption of minimally invasive concepts and the demand for enhanced recovery after surgery, spinal endoscopic surgery has developed rapidly. Facilitated by the advancement of endoscopic power tools and expandable cages, endoscopic fusion has become feasible. For patients with calcified discs or central spinal stenosis combined with bilateral lateral recess stenosis, uniportal coaxial endoscopic lumbar fusion has limitations, including potential inadequate decompression, risk of neural injury, and a steep learning curve, hindering its widespread clinical adoption and development. In 2012, Soliman (6) reported the use of a biportal irrigation endoscopic technique for LDH, achieving satisfactory clinical outcomes. Eum et al. (7) applied percutaneous biportal endoscopy for decompression of lumbar spinal stenosis in 2016, noting its excellent surgical field of view allowing simultaneous decompression of the central canal and bilateral lateral recesses. In 2017, Heo et al. (8) formally introduced the concept of Unilateral Biportal Endoscopy (UBE). Promoted primarily by Korean surgeons, biportal technology evolved rapidly. By establishing separate working and viewing portals, it enables decompression and fusion surgery with greater freedom of movement and a larger working space. The magnified endoscopic view and water medium provide a clearer visual field. Its relatively short learning curve, proven efficacy, minimal invasiveness, and significant short-term pain relief have led to its widespread application and expanding indications. In 2019, Heo and Park (9) first reported the Unilateral Biportal Endoscopic Lumbar Interbody Fusion (UBE-LIF, commonly referred to as UBE-TLIF) technique for lumbar degenerative diseases, demonstrating excellent clinical results. Our institution began performing UBE techniques in 2020, initially for simple discectomy, later expanding to Unilateral Laminotomy for Bilateral Decompression (ULBD) and endoscopic fusion.

Currently, surgery has become an important option after failed conservative treatment. The continuous development of minimally invasive techniques has led to the increasing clinical adoption of both UBE-TLIF and MIS-TLIF. While both techniques effectively decompress and stabilize the spine, comparative studies on postoperative recovery speed, degree of tissue trauma, and fusion efficiency require further validation. This study compares the long-term clinical outcomes of UBE-TLIF and MIS-TLIF in the treatment of LDH, aiming to provide evidence to inform clinical decision-making.

## 2 Materials and methods

### 2.1 Ethical approval and patient characteristics

This retrospective study analyzed 86 patients who underwent surgical treatment for single-level lumbar disc herniation at our hospital between August 2022 and August 2023. Among these patients, 42 underwent UBE-TLIF and 44 underwent MIS-TLIF. The surgeries were performed by two separate, specialized surgeon teams, each possessing extensive experience in their respective techniques. This study was approved by the Ethics Committee of Hefei First People’s Hospital (Approval no: 2024-2-67). Informed consent regarding surgical risks was obtained from all patients preoperatively. Patient demographics and characteristics are presented in Table 1. Statistical analysis using the *t*-test for age and the chi-square test for gender and surgical levels revealed that all *p*-values were substantially greater than the significance level of 0.05. This indicates no statistically significant differences between the two groups in key baseline characteristics, including age, gender distribution, and distribution of involved segments. The groups demonstrated strong comparability with balanced baseline characteristics, thereby minimizing potential confounding bias in subsequent analyses and establishing suitability for further comparative assessments of efficacy or prognosis.

**Table 1:** General information of two groups of patients.

General information	UBE-TLIF group	Mis-TLIF group		*p*
Patients total number	42	44		
Age	57.47 ± 9.89	56.75 ± 7.93	*t* = 0.372	0.71
Sex, male:female	23:19	27:17	*χ*^2^ = 0.385	0.535
Levels				
L4/5	26	29	*χ*^2^ = 0.149	0.699
L5/S1	16	15	*χ*^2^ = 0.163	0.703

Inclusion criteria: (1) Clinical manifestations of low back pain and lower limb radicular pain, aggravated by walking or exertion, with no significant improvement after 3 months of conservative treatment. (2) Radiological evidence of lumbar disc herniation accompanied by lumbar instability or refractory low back pain, where static radicular pain was mild but became significant upon weight-bearing/ambulation. (3) Absence of underlying conditions such as coagulation disorders.

Exclusion criteria: (1) Mismatch between symptoms/signs and radiological findings. (2) Unclear identification of the symptomatic level. (3) Concomitant other spinal pathologies, such as ankylosing spondylitis, spinal tumors, spinal fractures, or neurological diseases. (4) History of previous lumbar internal fixation surgery.

### 2.2 Surgical technique

#### 2.2.1 Ube-TLIF

**Figure 1:**
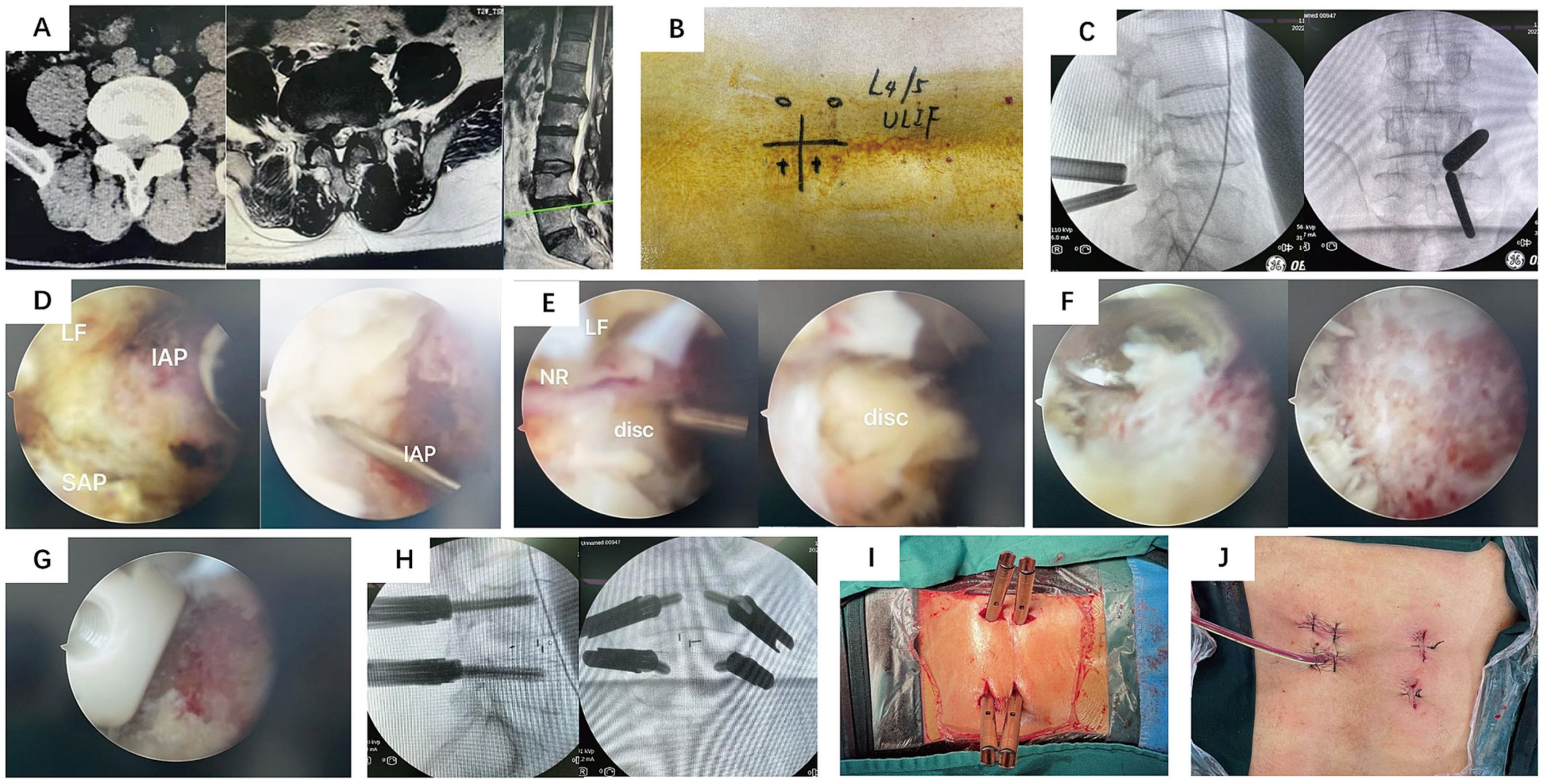
Representative Case 1. A 54-year-old male with low back pain and right lower limb radicular pain for 2 years, aggravated for 4 months. **(A)** Preoperative CT and MRI: right-sided L4/5 lumbar disc herniation compressing the nerve root and lateral recess stenosis. **(B)** Gross photo of the skin incisions. **(C)** Intraoperative fluoroscopic image for level localization. **(D–G)** Intraoperative endoscopic views showing decompression and cage insertion. **(H)** Intraoperative fluoroscopic image after pedicle screw placement. **(I,J)** Postoperative photos of the surgical wounds.

Following the induction of general anesthesia, the patient was positioned prone. The surgical field was routinely disinfected and draped. The wireless endoscope, along with intra-/extra-spinal radiofrequency (RF) probes and a powered drill system, were connected. Using the left-side approach as an example, an approximately 0.8 cm incision was made at the level corresponding to the inferior border of the pedicle of the superior vertebra at the target segment. This served as the endoscopic viewing portal. Another approximately 1.5 cm incision, oriented inferomedial to superolateral, was made at the level of the inferior vertebra’s pedicle, serving as the working portal. Extra-spinal RF ablation was used to clear soft tissues and establish the working space. After establishing the endoscopic portal, the superior and inferior articular processes and the lateral recess were addressed using either an endoscopic or an open osteotome and laminectomy rongeurs, potentially assisted by an endoscopic drill. Part of the ligamentum flavum and other soft tissues were removed to expose the intervertebral disc. The annulus fibrosus was incised, and disc material along with the cartilaginous endplates were removed using pituitary rongeurs, curettes, and shavers. The endoscope could be introduced into the disc space, allowing direct visualization and thorough preparation of the superior and inferior endplates by adjusting the scope’s angle. Under endoscopic guidance, local bone graft was impacted, and an interbody cage was implanted. The spinal canal was re-inspected to ensure adequate decompression and address any hidden compression. Finally, percutaneous pedicle screws and a connecting rod were inserted under fluoroscopic guidance to achieve segmental fixation. Please refer to Figure 1 for representative cases.

#### 2.2.2 MIS-TLIF group

**Figure 2:**
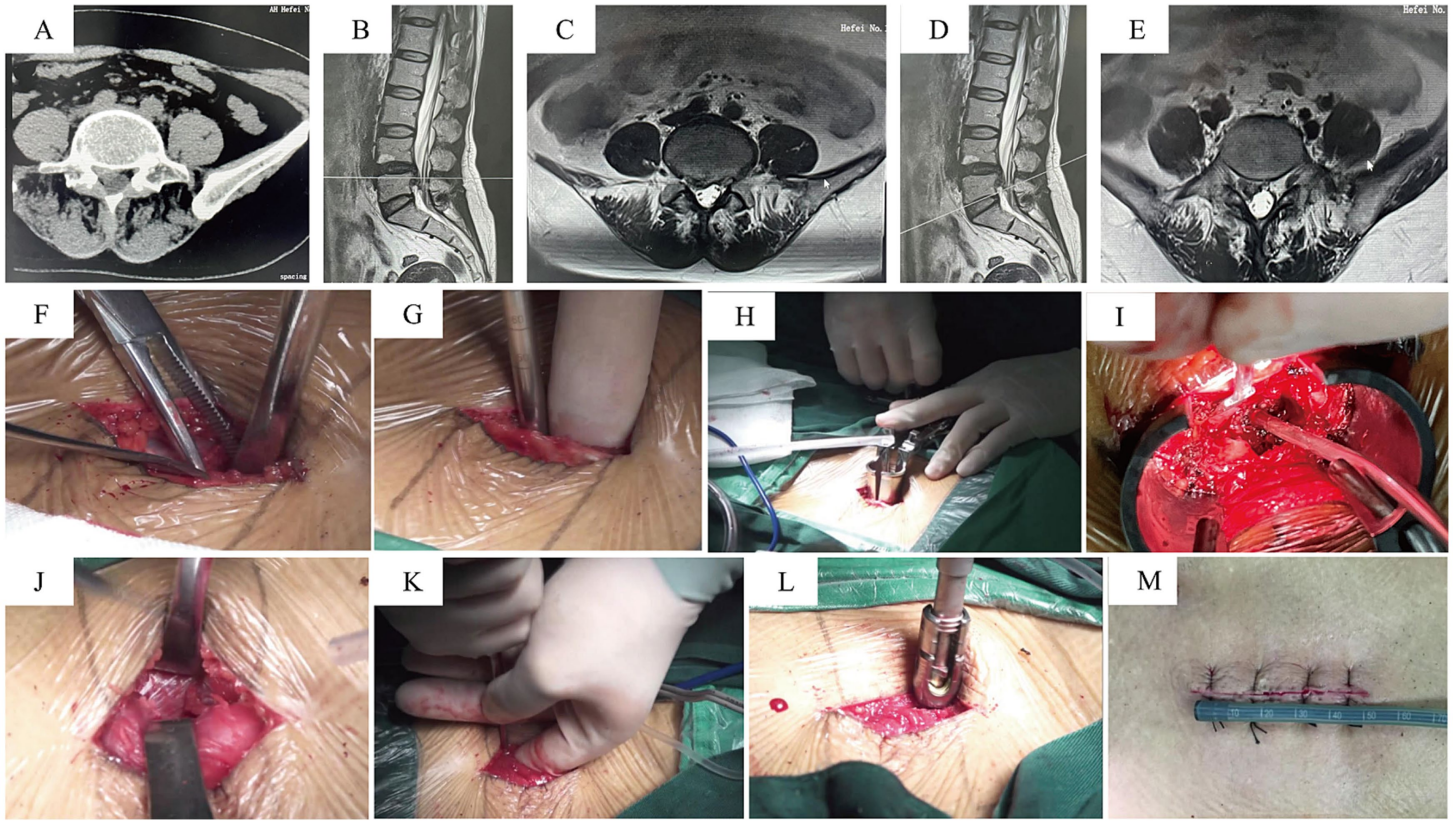
Representative Case 2. A 58-year-old female with low back pain and right lower limb numbness/pain for 2 years, aggravated for 2 months. **(A–E)** Preoperative CT and MRI: right-sided L4/5 lumbar disc herniation, migrated inferiorly, compressing the nerve root and causing foraminal stenosis. **(F–H)** A midline incision was used; the tubular retractor was inserted on the decompression side via an intermuscular plane. **(I)** View after completion of decompression. **(J–L)** Pedicle screws were placed on the contralateral side through the same midline incision via separate deep fascial incisions and an intermuscular approach. **(M)** Postoperative photo of the surgical wound.

After the induction of general anesthesia, patients were placed in the prone position. The surgical area was routinely disinfected and draped. A midline incision was made over the lumbar spine, exposing the thoracolumbar fascia. The fascia was incised approximately 2 cm lateral to the midline. The muscular plane was dissected through the intermuscular space, followed by the placement of a tubular retractor system. An osteotome and rongeurs were used to perform a facetectomy, removing the inferior articular process and part of the superior articular process. After partially removing the ligamentum flavum with rongeurs, the intervertebral disc was exposed. The disc material and soft tissues within the disc space were removed using pituitary rongeurs and curettes. Following endplate preparation and local bone grafting, an interbody cage was implanted. Pedicle screws were then inserted on the decompression side through the tubular retractor. The working channel was removed after confirming the absence of significant compression within the spinal canal. Contralateral pedicle screws and a connecting rod were inserted via a separate intermuscular approach to achieve segmental fixation. Please refer to Figure 2 for representative cases.

### 2.3 Efficacy evaluation

Patients were followed up clinically at 3 days, 1 month, 3 months, 12 months, and 24 months postoperatively. The following parameters were compared between the two groups: perioperative indicators (operative time, intraoperative blood loss, postoperative drainage volume, length of hospital stay), Visual Analogue Scale (VAS) scores for low back pain and leg pain preoperatively and at different postoperative time points, Oswestry Disability Index (ODI) scores preoperatively and at different postoperative time points, complication rates, surgical excellent-good rate (assessed by MacNab criteria), and fusion rate (evaluated by the Bridwell interbody fusion grading scale). Higher VAS scores (max 10) indicated more severe pain, while higher ODI scores (max 50) indicated worse quality of life. Clinical outcomes were assessed using the MacNab criteria. The fusion rate was evaluated at 12 months postoperatively based on follow-up CT scans using the Bridwell interbody fusion grading criteria.

### 2.4 Statistical analysis

All data were analyzed using SPSS statistical software (version 23.0). Measurement data are presented as mean ± standard deviation and were compared between groups using independent samples t-tests. Count data were compared between groups using the chi-square test or Fisher’s exact test. Ranked data were compared between groups using the rank-sum test. The significance level (*α*) was set at 0.05.

## 3 Results

### 3.1 Comparison of perioperative parameters between groups

All patients successfully underwent surgery. Details regarding operative time, intraoperative blood loss, length of hospital stay, postoperative fusion rate, and postoperative complications for both groups are presented in Table 2. The operative time in the UBE-TLIF group was significantly longer than that in the MIS-TLIF group (*p* < 0.05). For the UBE-TLIF group, the author further analyzed the operative time of the first 25 cases and the subsequent 17 cases. The operative times for these two subgroups were 155.34 ± 22.78 min and 121.27 ± 14.48 min, respectively. Although the smaller sample size of the latter 17 cases precluded a formal statistical comparison with the MIS-TLIF group’s operative time, the analysis suggests that operative time for UBE-TLIF can be significantly reduced with accumulated surgical experience. Conversely, the postoperative drainage volume in the UBE-TLIF group was significantly less than that in the MIS-TLIF group (*p* < 0.05).

**Table 2:** Perioperative parameters.

Perioperative parameters	UBE-TLIF group	Mis-TLIF group	*p*
Operative time (minute)	140.42 ± 16.02	92.15 ± 13.14	<0.0001
Postoperative drainage volume (mL)	65.79 ± 13.46	103.58 ± 12.56	<0.0001
Hospital stays (day)	9.74 ± 1.41	9.88 ± 1.19	0.775

### 3.2 Comparison of VAS scores for back and leg pain at preoperative and postoperative time points

The postoperative follow-up period for both groups was 24 months. Compared to preoperative values, VAS scores for back and leg pain and ODI scores at all postoperative time points were significantly reduced in both groups. Although the differences in VAS scores for back pain, leg pain, and ODI scores between the two groups were not statistically significant at most time points (*p* > 0.05), the UBE-TLIF group demonstrated lower VAS and ODI scores at all follow-up assessments. The difference observed at the 3-day postoperative mark was statistically significant (*p* < 0.05) (Table 3).

**Table 3:** Comparison of follow-up outcomes in group UBE-TLIF and group MIS-TLIF.

Relevant scores and grades	UBE-TLIF group	Mis-TLIF group	*p*
VAS of low-back pain			
Preoperative	6.94 ± 1.08	6.81 ± 1.06	0.667
3 days postoperative	1.84 ± 0.69	2.31 ± 0.79	0.045
1 month postoperative	1.11 ± 0.32	1.35 ± 0.49	0.066
6 month postoperative	0.68 ± 0.48	0.46 ± 0.51	0.144
12 month postoperative	0.26 ± 0.45	0.35 ± 0.49	0.563
24 month postoperative	0.27 ± 0.41	0.34 ± 0.45	0.642
VAS of leg pain			
Preoperative	7.16 ± 0.96	7.35 ± 1.13	0.56
3 days postoperative	0.63 ± 0.76	1.08 ± 0.93	0.096
1 month postoperative	0.53 ± 0.61	0.73 ± 0.60	0.271
6 month postoperative	0.42 ± 0.51	0.65 ± 0.56	0.16
12 month postoperative	0.23 ± 0.44	0.50 ± 0.51	0.114
24 month postoperative	0.25 ± 0.37	0.48 ± 0.26	0.125
ODI			
Preoperative	66.00 ± 3.94	65.15 ± 4.45	0.513
3 days postoperative	29.79 ± 3.33	30.81 ± 2.87	0.278
1 month postoperative	20.47 ± 2.52	20.08 ± 2.19	0.576
6 month postoperative	14.53 ± 2.06	15.19 ± 0.94	0.153
12 month postoperative	13.32 ± 2.53	14.27 ± 0.75	0.185
24 month postoperative			
MacNab			
12 month postoperative	27:13:2:0	28:14:2:0	0.88
Bridewell			
12 month postoperative	25:13:4:0	27:14:3:0	0.768

**Figure 3:**
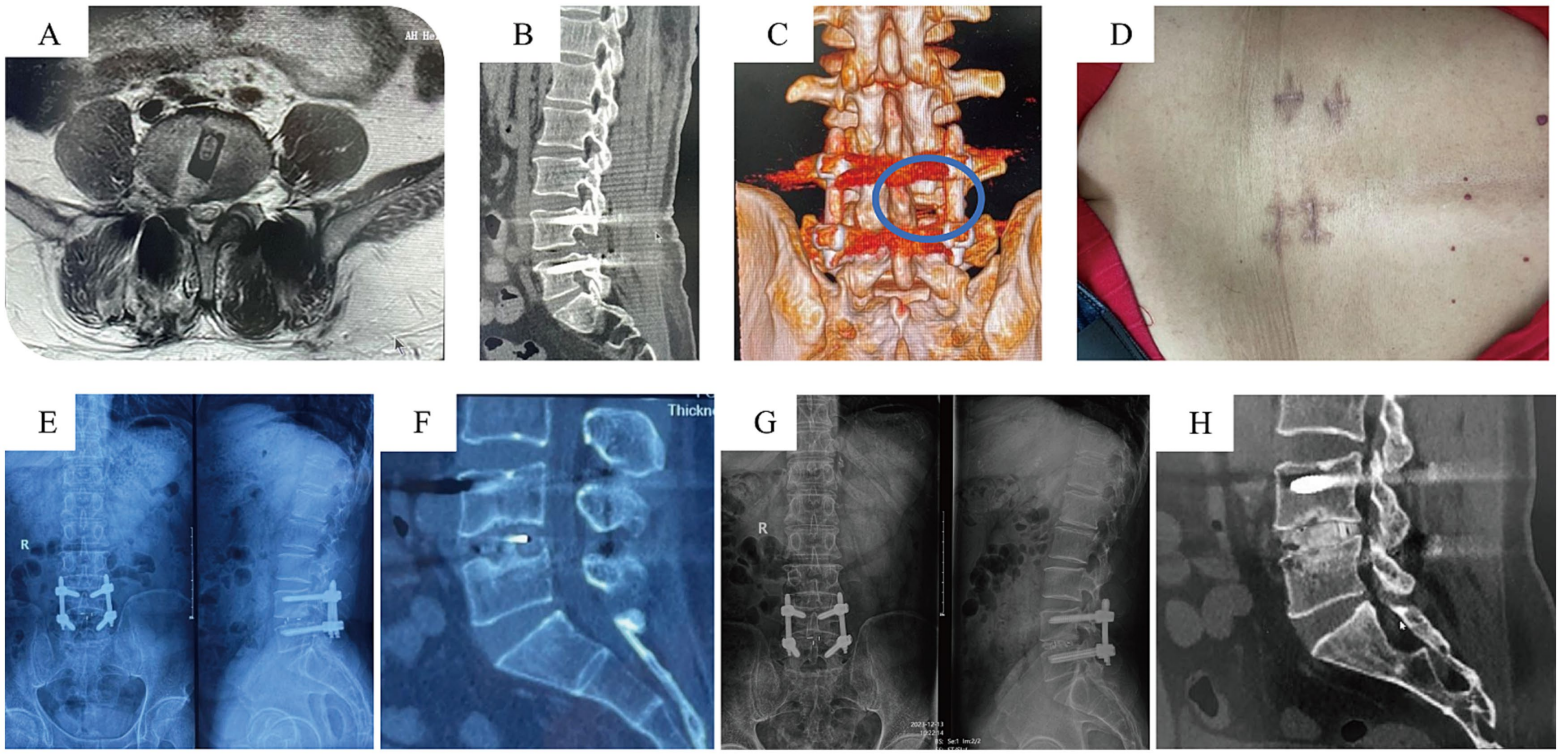
Postoperative follow-up data for UBE-TLIF Case. **(A–C)** Postoperative MRI indicating adequate decompression; CT showing the osteotomy surfaces of the superior and inferior articular processes. **(D)** Gross photo of the surgical wound. **(E,F)** X-ray and CT at 6 months postoperatively. **(G,H)** X-ray and CT at 2 years postoperatively indicating satisfactory fusion.

According to the MacNab criteria at 1-year postoperatively, the excellent and good rate for spinal stability was 95.2% in the UBE-TLIF group (27 patients rated as excellent, 13 as good, and 2 as fair) and 95.5% in the MIS-TLIF group (28 excellent, 14 good, and 2 fair) (Figure 3). The difference in outcomes based on the MacNab criteria was not statistically significant between the two groups (*p* > 0.05) (Table 3).

Based on CT scans at the 1-year postoperative follow-up and evaluated using the Bridwell interbody fusion grading scale, the UBE-TLIF group had 25 cases of Grade I fusion, 13 cases of Grade II, and 4 cases of Grade III, resulting in a fusion rate of 90.4%. The MIS-TLIF group had 27 cases of Grade I fusion, 14 cases of Grade II, and 3 cases of Grade III, yielding a fusion rate of 93.2%. The difference in fusion rates between the two groups was not statistically significant (*p* > 0.05) (Table 3).

### 3.3 Incidence, management, and follow-up of complications in both patient groups

No intraoperative complications, such as dural tears or nerve root injuries, occurred in either group. Each group reported one postoperative complication. In the UBE-TLIF group, one patient experienced transient contralateral hip soreness without radiating leg pain, sensory deficits, or motor weakness. After excluding issues related to screw placement, the symptoms resolved following treatment with hormones and mannitol. The patient’s VAS score was 1 at discharge, and the symptoms had completely resolved by the final follow-up, with no significant pain, sensory abnormalities, or motor deficits in the lower limbs. In the MIS-TLIF group, one patient developed minor marginal skin necrosis, characterized by whitening of the wound edges and slight exudate. Postoperative MRI showed no significant subcutaneous fluid collection or signs of infection, and inflammatory markers were not elevated. This was attributed to the small incision size and retractor traction during the procedure. The wound was managed with bedside debridement, excision of the necrotic skin edges, and suturing, resulting in delayed wound healing.

## 4 Discussion

With the rapid advancement of minimally invasive spine surgery techniques, lumbar fusion has entered an era where both endoscopic and tubular-assisted techniques coexist. This study, through a retrospective analysis, compared the clinical efficacy and perioperative indicators of UBE-TLIF and MIS-TLIF, two minimally invasive techniques, in treating single-level lumbar disc herniation, aiming to provide more detailed evidence-based guidance for clinical procedure selection.

### 4.1 Key findings: comparable long-term efficacy, divergent perioperative performance

The most significant finding of this study is that while both techniques achieved similar long-term clinical outcomes and fusion rates, they exhibited distinct characteristics in perioperative indicators and early postoperative recovery.

First, regarding core indicators of clinical efficacy, the 24-month follow-up data showed no statistically significant differences between the two groups in ODI score improvement, MacNab excellent-good rate, and CT-based Bridwell interbody fusion rates. This robustly confirms that UBE-TLIF and MIS-TLIF possess equivalent long-term effectiveness and safety in treating LDH, consistent with the findings of Heo, Yang, Kim, and others (9–11). However, the improvement in back and leg pain in the early postoperative period was superior in the UBE-TLIF group. The author attributes this to the lesser muscle damage under the endoscopic water medium and the washing away of inflammatory pain mediators by the irrigation fluid. Rathod’s (12) research confirmed that smaller surgical trauma leads to lower levels of inflammatory factors like IL and CRP, consequently facilitating faster recovery. Regarding the quantitative assessment of muscle injury extent between the two groups, the retrospective nature of this study precluded the availability of serial creatine kinase (CK) measurements and early postoperative MRI data for direct visualization of soft tissue disruption. Consequently, this observation requires validation in future prospective studies.

Second, the postoperative drainage volume was significantly less in the UBE-TLIF group compared to the MIS-TLIF group. This is likely primarily due to more meticulous hemostasis achieved under endoscopy. Given the high demand for a clear visual field in a water medium, pre-emptive hemostasis is particularly crucial. Active bleeding during endoscopic surgery can lead to a “red-out” screen, halting the procedure. Especially during endplate preparation, while significant bleeding occasionally occurs after disc space preparation in MIS surgery, it is generally avoided under the direct endoscopic vision and the effect of water pressure. Therefore, the thorough hemostasis inherent to endoscopic surgery results in less postoperative drainage.

Finally, the longer operative time in the UBE-TLIF group can be attributed to three main reasons, in our view: First, the surgeons in this study already had extensive experience (>400 cases) with MIS-TLIF, making them highly proficient. In contrast, UBE-TLIF was still in its early stages of adoption, requiring surgeons to adapt to the altered anatomical perspective of the endoscopic view. Second, the initial implementation of UBE-TLIF involves a learning curve (13–15). Even after overcoming this curve, although the biportal endoscope offers a large range of motion and a wide field of view, the limited diameter of the visual field still prevents some procedural steps from being completed in one motion. Steps like inferior articular process osteotomy and opening the lateral recess require careful execution. Furthermore, the procedure necessitates the use of both extra-spinal and intra-spinal radiofrequency probes for adequate and pre-emptive hemostasis throughout the process. Third, although open decompression instruments can be utilized, the efficiency under endoscopy is still lower than within an open tubular retractor, particularly during disc space preparation, which tends to require more time.

### 4.2 Technical limitations of traditional MIS-TLIF and modifications in this study

Since Professor Foley’s initial report in 2002, MIS-TLIF has been widely adopted due to its proven clinical efficacy and high fusion rates. However, it possesses the following drawbacks and limitations: (1) The air medium has high demands on lighting, making the management of complex bleeding within the spinal canal relatively challenging. (2) Decompression on the contralateral side is often inadequate due to constraints of the tubular retractor and instruments. Even tilting the operating table may not achieve sufficient decompression, failing to adequately expose the contralateral exiting nerve root and the entire lateral recess, and thus failing to accomplish complete bony decompression. Employing bilateral tubular retractors extends operative time and causes bilateral muscle damage (16), negating its minimally invasive advantage. (3) Although endplate preparation under the tube can be thorough and yield high fusion rates, it lacks direct visualisation, compromising the ability to properly protect and assess the endplates. This is particularly relevant for osteoporotic patients, where iatrogenic damage to the bony endplate raises the risk of long-term cage subsidence. (4) If the tubular retractor is suboptimally placed or soft tissue retraction is inadequate, better exposure may require excessive muscle destruction, potentially leading to early postoperative dead space and subsequent infection, or long-term scar formation and muscle degeneration contributing to chronic low back pain.

It is important to note that the MIS-TLIF performed in this study utilized a modified single midline incision, approximately 4 cm in length, with two separate incisions at the deep fascial layer. The tubular retractor was inserted on the decompression side via an intermuscular plane. Following decompression, pedicle screws were placed on the decompression side under direct vision. On the contralateral side, a percutaneous trajectory was established through an intermuscular approach for freehand screw placement. The specific technique involved palpating the junction of the superior articular process and the transverse process with a finger, fine-tuning to identify the entry point, and then sequentially preparing the screw path using a series of graded awls. This method requires the accumulation of substantial screw placement experience before it can be reliably employed. The modified MIS-TLIF technique used in this study offers several advantages: (1) Compared to dual incisions or a unilateral incision supplemented by two separate percutaneous incisions for contralateral screw placement, the single midline incision theoretically results in a shorter total incision length. Since both sides utilize an intermuscular approach, it causes less trauma and reduced blood loss. (2) This incision approach allows for the use of standard open pedicle screw systems, avoiding additional financial burden on patients associated with specialized percutaneous systems. (3) Direct visualisation during screw placement on the decompression side can reduce the number of intraoperative fluoroscopies required.

### 4.3 Operative details of UBE-TLIF

Regarding the technical details of UBE-TLIF surgery, our experience is as follows: After establishing the working space, an osteotome is used to remove the inferior articular process in pieces and the tip of the superior articular process en bloc. Pre-emptive hemostasis around the lateral joint capsule can be performed before resecting the tip of the superior articular process to avoid bleeding after its removal, which could obscure the endoscopic view and reduce efficiency. The advantage of the biportal technique lies in its sufficiently large working space, allowing the use of open instruments, thereby enhancing efficiency. During the early stages of adoption, angle disorientation can occur with the 30° endoscope. This can be mitigated by referencing the spatial relationship of the osteotome outside the body relative to the horizontal plane and patient position to determine its direction, akin to the spatial awareness in open surgery. The lateral recess is then addressed using pistol-grip rongeurs, with the resection extent reaching the inferior attachment of the ligamentum flavum on the lower vertebra’s lamina. At this point, the ligamentum flavum becomes mobile (“floating”) and no longer causes compression.

In our study cohort, the ligamentum flavum was preserved intraoperatively. By detaching its inferior attachment at the conjoined area (conner zone) of the lower vertebra’s lamina and its ventral attachment on the tip of the superior articular process (the foraminal ligament), the ligamentum flavum can be lifted away. This technique ensures it does not compress the thecal sac or nerve root canals. Furthermore, working under the protection of the reflected ligamentum flavum during decompression and disc space preparation minimizes irritation or traction on the nerve roots, potentially reducing intraoperative neural manipulation and even injury (17). For patients with concomitant central canal stenosis, the extent of bony decompression must reach the superior and inferior attachments of the ligamentum flavum to achieve complete mobilization (“floating”) of the entire ligamentum flavum, preventing inadequate decompression. For patients with simple lumbar disc herniation or predominant lateral recess stenosis, the cephalad decompression only needs to reach the level of the superior endplate of the involved disc space, avoiding unnecessary destruction of excessive bony structures.

### 4.4 Advantages and disadvantages of UBE-TLIF

The UBE-TLIF procedure enables fully endoscopic decompression of neural structures both inside and outside the spinal canal within a water medium. It integrates the advantages of tubular retractor technology, microscope technology, and uniportal endoscopy techniques, emphasizing the surgical philosophy of a “bloodless field and meticulous dissection.” This achieves the ideal goal of “performing open surgical procedures endoscopically.” The advantages of the UBE-TLIF technique are summarized as follows: (1) The biportal technique utilizes the natural interfascial plane of the multifidus triangle and creates a working space through water pressure. The viewing and working channels are independent, forming a triangular relationship. This provides a large operating space unrestricted by a tube, allows the use of open instruments and endoscopic power systems, and results in high working efficiency. (2) It represents the endoscopic transformation of traditional open surgery. Leveraging the magnified endoscopic view and extended operational range, it enables bilateral decompression through a unilateral approach, allowing simultaneous exploration of the contralateral traversing and exiting nerve roots. (3) The biportal operating mode allows for direct visualization during endplate preparation. The endoscope angle can be adjusted to inspect the condition of the superior and inferior endplates, helping to prevent iatrogenic injury to the bony endplate. This is particularly crucial for osteoporotic patients, where traditional MIS techniques carry a higher risk of endplate damage, which can lead to a series of complications such as cage subsidence affecting fusion, secondary foraminal stenosis, and implant loosening. Kim et al. (11) study demonstrated that vertebral endplates can be fully exposed under endoscopy, facilitating meticulous endplate preparation. The author’s experience also confirms that endplate handling is more intuitive and refined. However, it is undeniable that the UBE-TLIF technique has a learning curve. The author’s experience suggests the learning curve is typically overcome after approximately 30 cases, after which aspects like space establishment, decompression maneuvers, and operative time stabilize, consistent with literature reports (13–15). Nevertheless, challenges encountered during the early stages of implementation are undeniable, including prolonged operative time, loss of the working visual field, wrong-level surgery, the inability to repair dural tears endoscopically, and intracranial hypertension (18–21).

It is noteworthy that Unilateral Biportal Endoscopy (UBE) represents a comprehensive technical system rather than being confined to a single surgical approach. It can be adapted using various approaches to address different pathologies, such as the interlaminar, transforaminal, far-lateral, contralateral sublaminar, and contralateral oblique approaches. For pathologies related to the lumbosacral foramen, such as Far-out syndrome, which often involves obstruction by a high iliac crest making minimally invasive decompression challenging, the UBE technique—by simply modifying the incision placement—can achieve clear visualization of the foramen and extraforaminal regions. This minimizes excessive destruction of the facet joints and preserves spinal stability. Furthermore, as surgical techniques continue to mature and instruments improve, the indications and clinical applications of UBE are steadily expanding (22–27).

## 5 Limitations of this study

This study has several limitations. Firstly, it is a single-center retrospective analysis, which may introduce selection bias. Secondly, the relatively limited sample size might affect the statistical power to detect more subtle differences between the groups. Finally, intraoperative blood loss was not calculated in this study. This is because blood loss under an endoscopic fluid medium cannot be accurately measured by physical methods. Although some studies have used formulas based on changes in hematocrit for estimation (28, 29), the accuracy of this method is debated as it can be easily influenced by factors such as perioperative fluid administration. Consequently, intraoperative blood loss was not quantified in this research.

## 6 Conclusion

In summary, Unilateral Biportal Endoscopy (UBE) integrates the dual advantages of minimally invasive tubular techniques and endoscopic technology. It combines the working efficiency characteristic of an air medium with the minimal tissue trauma philosophy of a water medium, while simultaneously utilizing the endoscope to achieve an expanded field of view, enabling more extensive and precise decompression. The UBE-TLIF technique offers advantages such as a relatively manageable learning curve, a clear visual field, flexible instrument maneuverability, and direct visualization during endplate preparation. Its benefits are particularly evident in cases requiring bilateral decompression via a unilateral approach. This technique demonstrates confirmed long-term clinical efficacy in the treatment of lumbar disc herniation. Although challenges such as prolonged operative time are encountered during the initial implementation phase, the operative duration can be significantly reduced with accumulated surgical experience, supporting its broader clinical adoption.

## Data Availability

The raw data supporting the conclusions of this article will be made available by the authors, without undue reservation.
